# C1q–HA Matrix Regulates the Local Synthesis of Hyaluronan in Malignant Pleural Mesothelioma by Modulating HAS3 Expression

**DOI:** 10.3390/cancers13030416

**Published:** 2021-01-22

**Authors:** Romana Vidergar, Andrea Balduit, Paola Zacchi, Chiara Agostinis, Alessandro Mangogna, Beatrice Belmonte, Micaela Grandolfo, Francesco Salton, Marco Biolo, Fabrizio Zanconati, Marco Confalonieri, Roberta Bulla

**Affiliations:** 1 Department of Life Sciences, University of Trieste, 34127 Trieste, Italy; romana_vidergar@immunol.a-star.edu.sg (R.V.); pzacchi@units.it (P.Z.); rbulla@units.it (R.B.); 2 Institute for Maternal and Child Health, IRCCS Burlo Garofolo, 34134 Trieste, Italy; cagostinis@units.it (C.A.); alessandro.mangogna@burlo.trieste.it (A.M.); 3 Tumor Immunology Unit, Department of Health Sciences, University of Palermo, 90133 Palermo, Italy; beatrice.belmonte@unipa.it; 4 International School for Advanced Studies (SISSA), 34136 Trieste, Italy; micaela.grandolfo@sissa.it; 5 Department of Medical, Surgical and Health Science, University of Trieste, 34129 Trieste, Italy; francesco.salton@studenti.units.it (F.S.); marco.biolo@asugi.sanita.fvg.it (M.B.); fabrizio.zanconati@aots.sanita.fvg.it (F.Z.); marco.confalonieri@asugi.sanita.fvg.it (M.C.)

**Keywords:** malignant pleural mesothelioma, cancer, hyaluronic acid, immune system, complement system, C1q, tumor microenvironment, hyaluronan synthases, HAS3

## Abstract

**Simple Summary:**

Malignant pleural mesothelioma (MPM) is a rare and aggressive tumor characterized by poor prognosis due to late diagnosis and the absence of efficient first-line treatments. Hyaluronic acid (HA) and the complement protein C1q represent two pivotal players in the MPM tumor microenvironment by acting in association with effects on cancer cell adhesion, migration and proliferation. The aim of the current study is to prove HA production by MPM primary cells and to understand whether HA metabolism modulation could be considered a potential target for future therapeutic approaches in MPM.

**Abstract:**

Increased hyaluronic acid (HA) production is often associated with cancer progression. In malignant pleural mesothelioma (MPM), HA is found at elevated levels in pleural effusions and sera of patients, and it has been widely debated whether MPM cells are able to produce HA by themselves or through the release of growth factors stimulating other cells. Another key component of the MPM microenvironment is C1q, which can act as a pro-tumorigenic factor favoring cell adhesion, migration and proliferation. The aim of the current study was to prove that MPM primary cells are able to synthesize HA and to inquire the stimulus given by C1q–HA matrix to HA synthesis. We confirmed the presence of a HA coat and cable-like structures around MPM primary cells, as well as an intracellular pool, mainly localized in the cytoplasmic and perinuclear region. After evaluating HA synthase (HAS) enzymes’ basal expression in MPM primary cells, we found that C1q bound to HA was able to impinge upon HA homeostasis by upregulating HAS3 both at the mRNA and the protein levels. High expression of *HAS3* has been correlated with a shorter life expectancy in MPM by bioinformatical analysis. These data confirmed that C1q bound to HA may exert pro-tumorigenic activity and identified HAS3 as a potential target in MPM.

## 1. Introduction

Malignant pleural mesothelioma (MPM) is a rare and aggressive kind of cancer arising from the mesothelial cells of the pleura. The principal and widely acknowledged risk factor associated with MPM is asbestos exposure, with a latency period varying from 20 to 50 years [1]. Non-specificity of symptoms, late diagnosis and absence of efficient first-line therapeutic options ensure an almost certainly poor prognosis [2].

The unique MPM tumor microenvironment, in addition to genetic abnormalities accumulating in mesothelial cells, is responsible for tumor capacities of migration, invasion and metastasis [3,4]. Within this context, the extracellular matrix (ECM) and in particular one of its components, hyaluronic acid (HA), plays a pivotal role in MPM progression [5,6]. HA, as a member of the glycosaminoglycan (GAG) family, has a relatively simple structure and is abundantly produced throughout all mammalian species [7]. HA synthesis, unlike of other GAGs, occurs in the plasma membrane and hyaluronan synthases (HAS1, HAS2 and HAS3) are the integral membrane proteins responsible for its production, showing different enzymatic activity [8,9]. HA synthase enzymes are glycosyltransferases exerting their catalytic activity on the inner side of plasma membrane, where they synthetize large and linear polymers of the repeating disaccharide structure of hyaluronan by adding alternately the two precursors, uridine diphosphate (UDP)-N-acetylglucosamine and UDP-glucuronic acid, to the reducing terminus of the chain [10].

Significantly increased levels of HA production are often associated with human and animal tumors [11]. The contribution of HA to tumor progression is based on two fundamental mechanisms: the first is the HA metabolism, including the regulation of HA metabolic enzymes that define the amount and size of HA [12]; the second one is the binding of HA to cell membrane receptors that activate signaling pathways and modulate cell functions [13]. Interestingly, the molecular weight of HA may affect its biological properties [14].

HA synthesis by human MPM cells is an issue that has been widely discussed over the course of time. MPM is commonly associated with high content of HA; in particular, it can be found at elevated levels in pleural effusions [15] and sera [16] of patients. Nevertheless, several studies have suggested that the increased HA synthesis reported in patients affected by MPM is mostly due to the release of growth factors from tumor cells that may stimulate other cells to produce HA [17,18].

Another key component of tumor microenvironment is the complement system and, specifically, its recognition component of the classical pathway, namely C1q. This macromolecular complex has been shown to act as a tumor-promoting factor by favoring adhesion, migration and proliferation of cancer cells [19]. C1q has been previously demonstrated to bind a wide range of target ligands within the ECM and a particularly strong interaction has been evidenced with HA, enhancing even more tumor-progressing behaviors [20]. Furthermore, we previously demonstrated the high abundance of C1q within the MPM microenvironment [20].

In the current study, we sought to prove that MPM primary cells isolated from patients are able to synthesize hyaluronic acid by themselves, also providing some information about its intracellular localization. In addition, we inquired the stimulus given by the C1q–HA matrix to HA synthesis.

## 2. Results

### 2.1. MPM Cells Are Able to Synthesize Endogenous HA by Themselves

**Figure 1:**
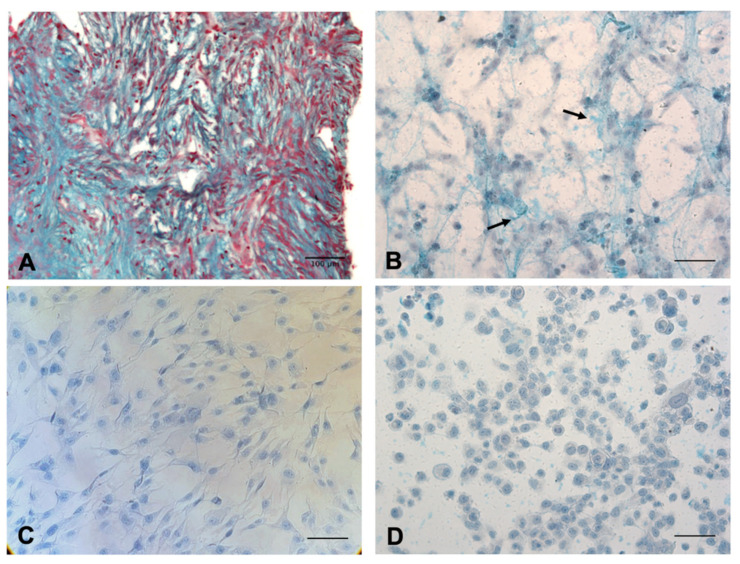
(**A**) Hyaluronic acid (HA) presence in the malignant pleural mesothelioma (MPM) tumor microenvironment. Histochemical staining with Alcian Blue highlighted HA distribution in MPM tissue sections; in particular, the staining was visible in tumor-associated stroma. Nuclei were stained with Fast Red. Magnification, 200×, scale bar, 100 µm. (**B**–**D**) MPM primary cells are able to produce HA by themselves, as compared to normal mesothelial cells Met5A. Alcian blue staining in MPM primary cells highlighted the presence of HA around the cells (indicated by black arrows) (**B**). Hyaluronidase treatment allowed the removal of HA staining (**C**). As a further control, Met5A cells, a normal mesothelial cell line, do not show the capability to produce HA (**D**). Original magnification, 200×, scale bar, 50 µm.

We initially aimed at investigating HA presence in the MPM tumor microenvironment by histochemical analysis of paraffin-embedded MPM tissue samples stained with the Alcian blue solution, a cationic dye used for the detection of acidic glycosaminoglycans. As shown in Figure 1A, a strong positivity for HA could be detected in particular in tumor-associated stroma.

Once we had demonstrated HA presence at the tissue level, we proceeded further by assessing whether MPM primary cells possessed the capability of synthesizing endogenous HA by themselves. After methanol-based fixation, MPM primary cells were stained with the Alcian blue dye, highlighting the presence of HA cables around MPM primary cells (Figure 1B). To confirm the cables as being composed of HA, a hyaluronidase treatment (500 units/mL) was performed. In Figure 1C, it can be noticed that upon hyaluronidase addition, the cables around the cells were completely lost. As a further control, the same experiment was carried out in Met5A, a mesothelial cell line derived from non-cancerous individuals; furthermore, in this case, we could not detect any blue staining (Figure 1D).

**Figure 2:**
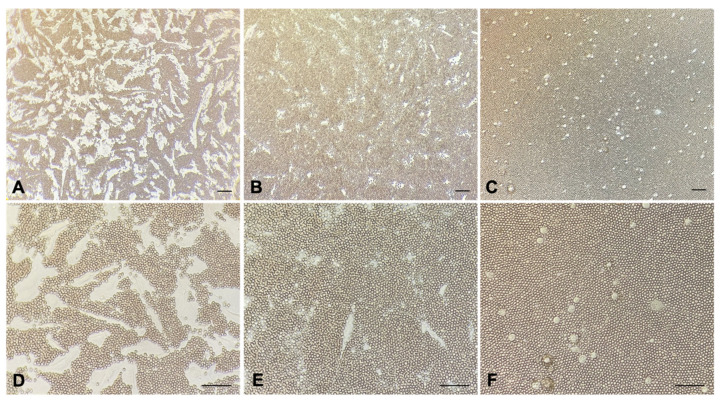
Red blood cell (RBC) exclusion assay in MPM primary cells with or without hyaluronidase treatment and in Met5A cells. Fixed RBCs were added onto MPM primary cells in the culture, highlighting the presence of a HA pericellular coat around the cells (**A**,**D**); hyaluronidase treatment (**B**,**E**) was performed to confirm the coat as being composed of HA. As an additional control, the same experiment was carried out with Met5A (**C**,**F**). Original magnification, 100× (**A**–**C**), 200× (**D**–**F**), scale bar, 50 µm.

A further investigation about HA production was obtained by red blood cell (RBC) exclusion assay. MPM primary cells were seeded at low confluence and allowed to deposit around them a HA pericellular coat for 48 h. When fixed RBCs were added onto the cells in the culture and enabled to settle down, only MPM primary cells displayed a wide halo that excluded fixed erythrocytes (Figure 2A,D). This halo was not observed neither on seeded Met5A (Figure 2C,F) nor upon hyaluronidase treatment of MPM primary cells (Figure 2B,E).

### 2.2. Detection of Intracellular HA and HA Cables in MPM Cells

**Figure 3:**
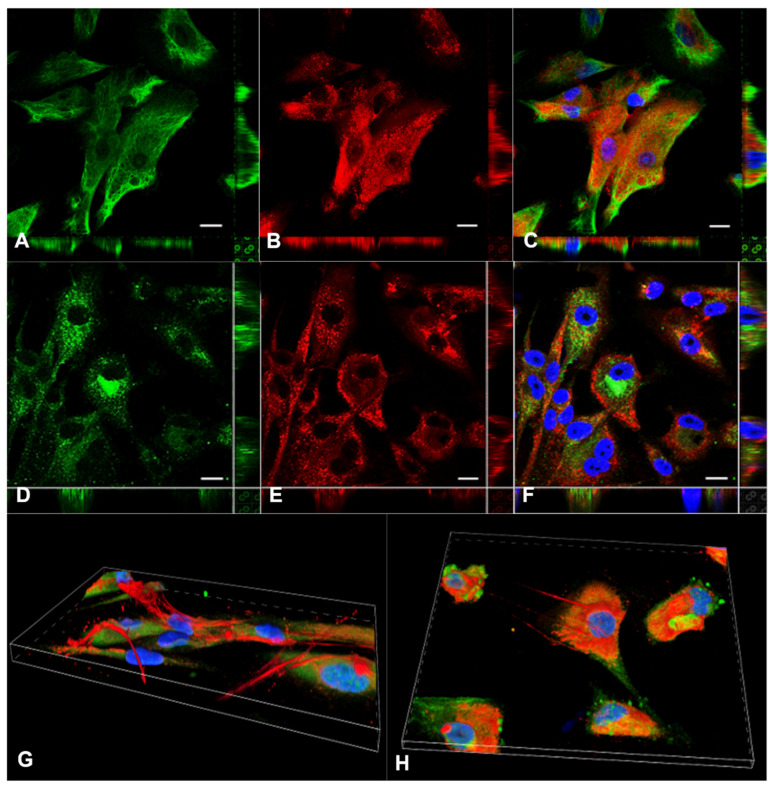
(**A**–**C**) HA synthesis by primary MPM cells and its intracellular distribution. MPM primary cells were fixed, permeabilized and co-stained for vimentin (**A**) in order to visualize cell morphology; and for HA binding protein (HABP) (**B**) in order to detect HA presence inside the cells. Intense HA staining inside the cytoplasm of MPM primary cells was clearly detected and illustrated by the reported z-projections. The merge (**C**) allowed the visualization of intracellular HA staining as vesicular or globular structures prominently concentrated in the perinuclear region of cells. Nuclei were stained in blue. The presence of an HA pericellular coat could not be detected because of the use of fixatives. Scale bar: 10 µm. (**D–F**) Endocytosis of exogenous HA by MPM primary cells. Fluorescein-labeled (HA-Fl) (10 µg/mL) was supplied to MPM primary cells in the culture to be taken up during overnight (ON) incubation (**D**). Cells were then fixed and stained with HABP for endogenous intracellular HA detection (**E**). The two pools do not overlap (**F**). Nuclei were stained in blue. Scale bar: 10 µm. (**G**,**H**) 3D reconstruction of the distribution of HA produced by MPM primary cells. Detection of HA cables, protrusions that connect adjacent cells, was observed in particular in the perinuclear region.

In order to obtain further insights about endogenous HA synthesis by MPM cells, we took advantage of hyaluronan-binding protein (HABP) as a probe for HA detection. HA in fact is not immunogenic, but it can be specifically recognized by HABP which typically comprise the G1 domain of aggrecan or a combination of G1 and the cartilage link protein [21]. Double-staining experiments were therefore performed using a biotinylated HABP probe (5 μg/mL) for HA detection together with an anti-vimentin antibody required to appreciate cell morphology. HABP binding to HA was then revealed upon incubation with Cy3-conjugated streptavidin. As a control, MPM primary cells were treated with commercially available hyaluronidases with the aim to selectively degrade the pericellular halo MPM primary cells are used to deploy around them. Interestingly, hyaluronidase-treated and untreated MPM primary cells showed only intense hyaluronan staining localized in the cytoplasm, as illustrated by the reported z-projections (Figure 3A–C). This intracellular HA signal was organized as vesicular/globular structures dispersed within the whole cellular volume and predominantly evident also in the perinuclear region. The extracellular hyaluronan coat could not be detected since it is easily lost during the fixation procedure, therefore impeding appreciation of the differences between the cells treated or untreated with hyaluronidases (data not shown).

To investigate whether an endocytosed extracellular pool of HA could be the main source for intracellular HA, we filled the whole endocytic system upon incubating the cells ON with 10 μg/mL of fluorescein-labeled HA (HA-Fl) supplied in the culture medium. The following day the cells were fixed and labeled for endogenous HA using the HABP probe. As shown in Figure 3D–F, the signals deriving from the two HA pools, the one synthesized by MPM primary cells and deployed extracellularly versus the exogenously supplied fluorescein-labeled fraction, did not overlap, with the fluorescein signal restricted to large endosomal vesicles.

3D reconstruction of the distribution of HA produced by MPM primary cells allowed us to identify the presence of HA cables. From the 3D images taken by confocal microscopy, we could confirm what was previously described in the literature [22], since it was possible to clearly visualize some HA protrusions (Figure 3G,H) to connect adjacent cells, in particular the perinuclear region.

### 2.3. Intracellular Localization of HA in Cell Organelles

**Figure 4:**
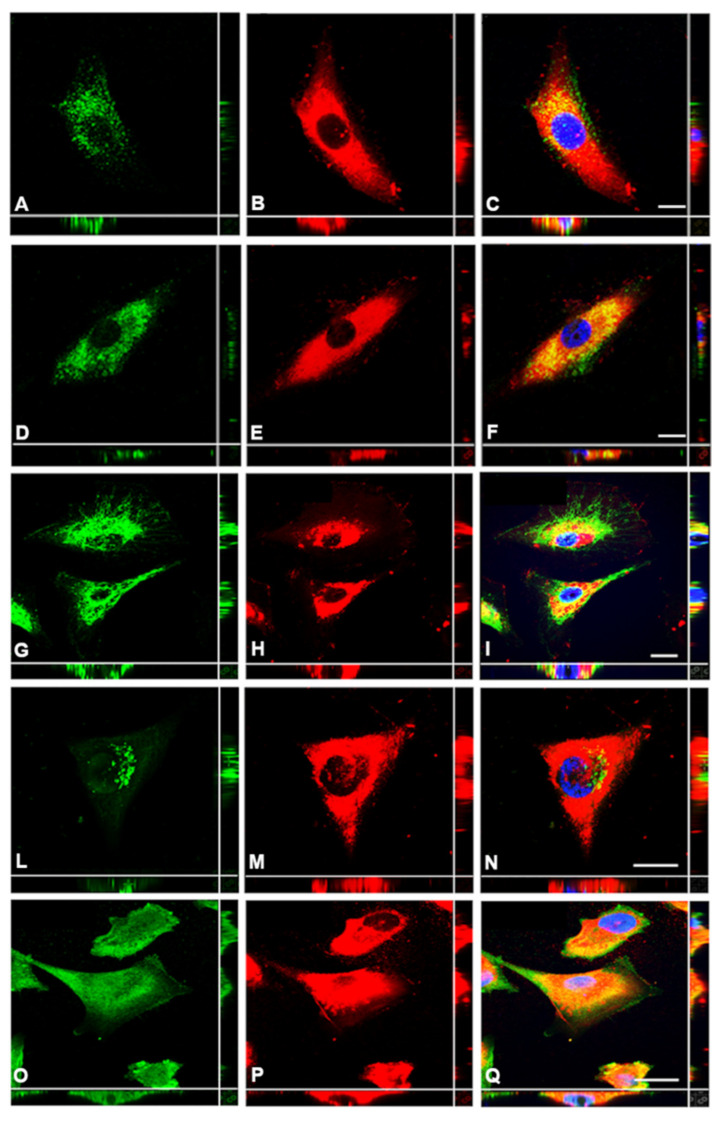
Intracellular HA distribution along endocytic and secretory pathways. MPM primary cells were fixed, permeabilized and co-stained for the HA-binding protein (**B**,**E**,**H**,**M**,**P**) and markers of the endocytic pathway, EEA1 (**A**) and Lamp-1 (**D**), as well as for secretory pathways, calnexin (**G**), TGN38 (**L**) and Rab11A (**O**). The overlap allowed excluding colocalization along the endocytic pathway (**C**,**F**), low intensity of colocalization for calnexin (**I**) and TGN38 (**N**) and stronger intensity for Rab11A (**Q**). Scale bar: 10 µm.

To achieve clearer understanding of the intracellular distribution of endogenous HA, in particular to distinguish between the possibility of detecting HA mainly sequestered within intracellular organelles/vesicles or freely dispersed into the cytosol, we performed a series of immunofluorescence experiments by colabeling HA with specific markers of the endocytic and secretory pathways (Figure 4). Early endosome antigen 1 (EE1A), lysosomal-associated membrane protein 1 (LAMP1) and Ras-associated binding protein 11A (Rab11A) were chosen as target antigens for early endosomes, lysosomes and recycling endosomes, respectively. Calnexin and Trans-Golgi network membrane protein 38 (TGN38) were used instead to label endoplasmic reticulum (ER)-to-Golgi and the Trans-Golgi network (TGN) compartments of the secretory pathway. The detection of just a partial colocalization with calnexin and Rab11A seems to suggest anyway that HA is mainly localized in the cytoplasm of the MPM tumor cells.

### 2.4. Basal Expression of HAS Enzymes in MPM Tissue Samples and MPM Cells

**Figure 5:**
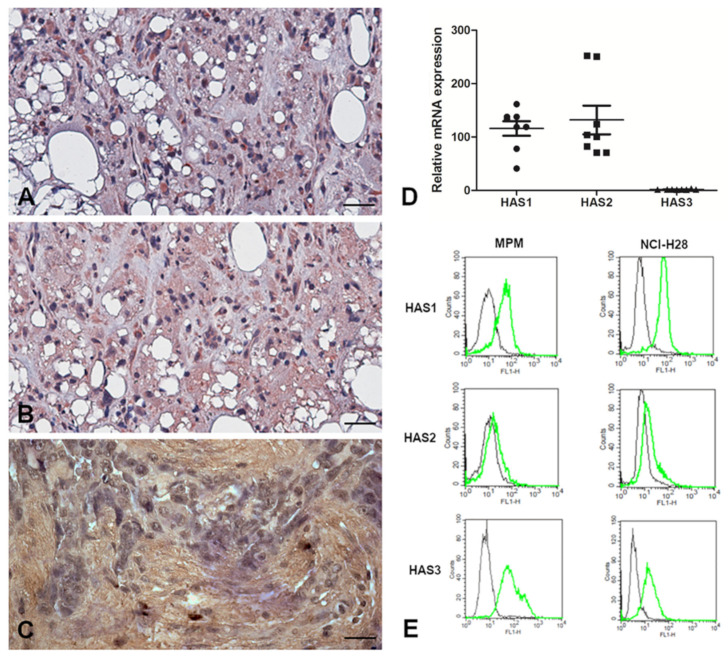
Characterization of HAS enzyme expression at the tissue level in MPM sections and in MPM cells. (**A**–**C**) Immunohistochemical analysis of HAS enzymes in MPM tissues. MPM sections were probed for the detection of HAS1 (**A**), HAS2 (**B**) and HAS3 (**C**) expression at the tissue level, showing cytoplasmic and membrane presence of all the three HAS isoforms within the tumor region. Staining was revealed using a polymer detection kit (Novocastra, Leica Biosystems) and the AEC (3-amino-9-ethylcarbazole, Dako, Denmark) or DAB (3,3′-diaminobenzidine) substrate chromogen. Slides were counterstained with Harris Hematoxylin (Novocastra, Leica Biosystems). Magnification, 200×, scale bar, 100 µm. (**D**) Basal mRNA expression level of HAS enzymes in MPM primary cells using quantitative real-time PCR. Higher expression of *HAS1* and *HAS2* was highlighted. TATA box-binding protein (*TBP*) was used as a housekeeping gene to normalize gene expression results. Data were expressed as the means of experiments performed on eight different MPM populations in triplicates ± SEM. (**E**) Basal protein expression level of HAS enzymes in MPM cells using cytofluorimetric analysis. MPM cells were stained for HAS1, HAS2 and HAS3 and the fluorescence intensity of the cells incubated with primary antibodies (green line) was compared with unrelated staining (black line). All three enzymes were expressed by MPM primary cells. Basal protein expression level of HAS enzymes was also evaluated in NCI-H28, a human mesothelioma cell line. HAS enzyme expression in NCI-H28 cells appeared comparable to the one of MPM primary cells.

As the first step towards the identification of the expression level and the intracellular distribution of the main enzymes involved in HA synthesis, namely HAS1, HAS2 and HAS3 isoforms, we performed immunohistochemical labeling on paraffin-embedded sections derived from MPM tissues (Figure 5A–C) with isoform-specific antibodies. All three HAS isoforms had similar cytoplasmic and membrane-associated expression within the tumor region.

In order to investigate the basal expression levels of the hyaluronan synthases HAS1, HAS2 and HAS3 in primary cells, we proceeded then with quantitative real-time PCRs of the total mRNA samples extracted from freshly isolated MPM cells. As shown in Figure 5D, *HAS1* and *HAS2* resulted in the predominantly expressed synthetic enzymes in all the eight MPM primary cell populations, highlighting interesting variability in all the cases. On the other hand, *HAS3* expression was very low when considering the mRNA level. To evaluate expression of the three isoforms at the protein level, we performed cytofluorimetric analysis. As shown in Figure 5E, the cells were positively stained for all three HAS enzymes. The same experiment was also repeated on the NCI-H28 cell line derived from the human mesothelioma epithelioid histotype, confirming the expression pattern similar to that observed in primary MPM cells.

### 2.5. Modulation of HAS Enzyme Expression after HA and C1q Treatment of MPM Cells

**Figure 6:**
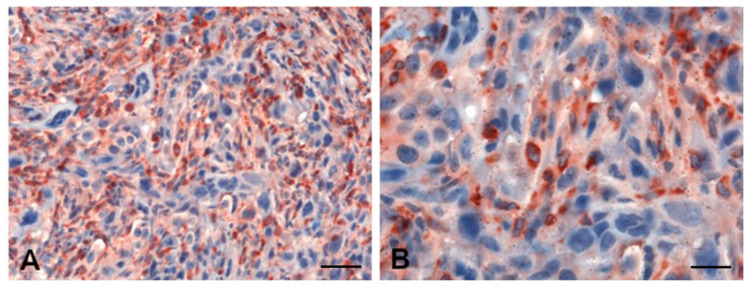
C1q expression within the MPM tumor microenvironment. Immunohistochemical analysis of C1q expression in MPM specimens highlighted massive presence of C1q in the tumor stroma and in the cytoplasm of monocytoid cells. Staining was revealed using a polymer detection kit (Novocastra, Leica Biosystems) and the AEC (3-amino-9-ethylcarbazole, Dako, Denmark) substrate chromogen. Slides were counterstained with Harris Hematoxylin (Novocastra, Leica Biosystems). Magnification: (**A**) 200×, scale bar, 100 µm; (**B**) 400×, scale bar, 50 µm.

Assessment of the basal HAS expression was followed by the evaluation of the influence exerted by HA and C1q on HAS modulation. Having further demonstrated a massive presence of HA within the MPM tumor microenvironment, we proceeded with an immunohistochemical analysis for the detection of C1q in MPM tissues. As shown in Figure 6, a strong positivity for C1q was detected in MPM specimens, mainly expressed in the cytoplasm of intratumoral inflammatory cells, in the tumor stroma and in small vessels, but completely absent in neoplastic cells.

**Figure 7:**
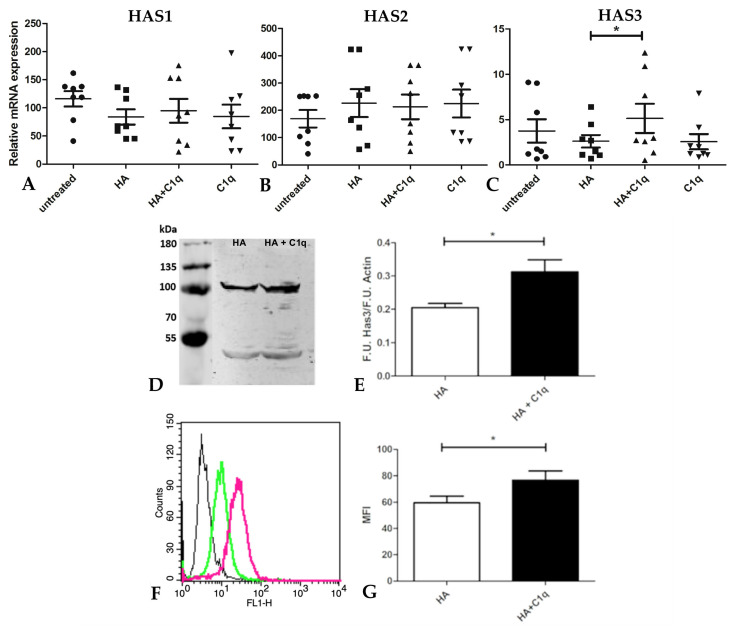
HAS modulation in MPM primary cells after treatment with C1q and/or HA. (**A**–**C**) C1q and/or HA were immobilized as a matrix and MPM primary cells, after ON incubation, were analyzed by quantitative real-time PCR, highlighting upregulation of *HAS3* mRNA expression level. Data were expressed as the means of experiments performed on eight different MPM populations in triplicates ± SEM, * *p* < 0.05. (**D**,**E**) Western blot analysis of HAS3 expression in MPM primary cells seeded onto HA or the HA and C1q matrix. Cell lysates were separated by SDS-PAGE and, after the transfer process, the membrane was probed with the α-HAS3 primary antibody and a LI-COR IRDye secondary antibody. Fluorescence intensity was identified using an Odyssey^®^ CLx near-infrared scanner (LI-COR Biosciences, Lincoln, NE, USA). Image acquisition, processing and data analysis were performed with Image Studio ver 5.2 (LI-COR Biosciences). Β-actin was used to normalize the results, * *p* < 0.05. (**F**,**G**) Cytofluorimetric analysis of HAS3 expression after the HA (green line) or the HA and C1q (magenta line) treatment. Fluorescence intensity of the cells incubated with the HAS3 primary antibody was compared with unrelated staining (black line). Upregulation of HAS3 at the protein level was confirmed in MPM cells, * *p* < 0.05.

Based on the observations about their presence in the MPM tumor microenvironment, HA and C1q were initially supplied as soluble “ligand” treatments, but this approach proved quite ineffective and unsatisfactory (Figure S1). Therefore, we evaluated the possibility of immobilizing HA in the presence or absence of C1q as a matrix. MPM primary cells were therefore seeded on these matrixes and total mRNA was purified after ON incubation with HA and/or C1q. Based on quantitative real-time PCR analysis, the C1q–HA matrix seemed to significantly increase the *HAS3* expression level as compared to HA alone, whereas *HAS1* and *HAS2* were not modulated by HA and/or C1q stimuli (Figure 7A–C).

In order to also confirm HAS3 modulation at the protein level, the cells were seeded at the same coating conditions and HAS3 expression was evaluated by Western blot and cytofluorimetric analyses. Both Western blot analysis (Figure 7D,E) and cytofluorimetric assay (Figure 7F,G) confirmed upregulation of HAS3 expression when the cells were seeded onto the C1q and HA matrix.

### 2.6. Clinical Significance of HAS mRNA Expression in MPM

**Figure 8:**
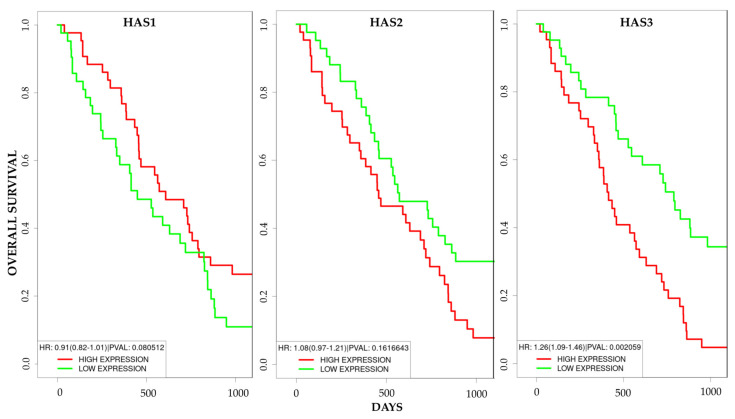
Patients’ clinical outcomes on the basis of *HAS1*, *HAS2* and *HAS3* mRNA expression in MPM cancer. *HAS1* and *HAS2* expression is not correlated to MPM patients’ survival rate, whereas high *HAS3* mRNA expression is correlated to a lower survival rate of mesothelioma patients as determined by using the PROGgene V2 database. *p* < 0.01.

In order to understand whether HAS3 could serve as a potential prognostic target in human MPM, we took advantage of the bioinformatical survival analysis database PROGgeneV2, drawing from The Cancer Genome Atlas Mesothelioma (TCGA-MESO) dataset for the generation of a survival plot. Based on this dataset, we observed that, whilst *HAS1* and *HAS2* (Figure 8) expression seemed not to be correlated to patients’ survival, the *HAS3* expression level was negatively correlated to life expectancy in MPM patients (Figure 8): survival rate of patients with high *HAS3* expression was lower than of the ones with low expression (*p* < 0.01), as illustrated in The Cancer Genome Atlas (TCGA dataset).

## 3. Discussion

Significantly increased levels of HA are often associated with human and animal tumors [23]. During tumor invasion, changes of the tumor ECM occur due to proteolytic cleavage of structural proteins, and it is common to find a more hydrated ECM enriched in HA [24].

MPM is commonly associated with a high content of HA; in particular, it can be found at elevated levels in pleural effusions and sera of patients [15,16], showing also higher HA positivity levels around tumor cells as compared to metastatic adenocarcinoma [25]. The evidence of strong HA presence in the MPM tumor microenvironment was also confirmed by our histochemical analysis performed both on tissue sections and isolated primary MPM cells, highlighting intense HA staining in particular in the tumor stroma.

Despite its undeniable presence within the MPM microenvironment, HA production by human MPM cells is an issue that has been widely debated over the last decades, leading to contradictory results. An experimental report by Asplund and colleagues supported the notion that MPM cell lines produce only a small amount of HA as compared to normal mesothelial cells which synthesize large quantities of HA; conditioned media from human MPM-derived cell lines were shown to promote HA synthesis in vitro by fibroblasts and mesothelial cells due to secreted growth factors or other mediators [17]. Similar results have also been shown in other types of malignancy [26,27,28].

RBC exclusion assay and Alcian blue staining performed by our group revealed the presence of an HA pericellular coat surrounding MPM cells isolated from diagnostic biopsy and seeded at low density. This capacity was totally absent in Met5A normal mesothelial cells, proving their inability to synthesize HA by themselves. In addition, based on double staining immunofluorescence assays, where HA detection by the HABP probe was associated with several markers for the endocytic and secretory pathways, and on HA-Fl endocytosis experiments, we proved the ability of human MPM primary cells to synthesize large amounts of HA not enwrapped into organelles/vesicular structures but freely dispersed into the cytosol.

Despite observing the presence of secreted HA as a well-described component of the extracellular matrix and its organization in HA cables for cell-to-cell communication and cell migration [22,29], a considerable pool of the HA located intracellularly was also detected. It appeared as round vesicular or globular structures filling the whole cell volume and, in most cases, clustered in the perinuclear region of the cell. Indeed, the presence of HA in the nuclear periphery and/or nucleoli has already been well-described [30] despite raising several still unanswered questions about the functional role played by cytosolic HA. It has also been reported that HA is synthesized in large amounts by mitotic cells, suggesting an involvement of HA in growth regulation and mitosis [31,32]. The finding of such a strong presence of intracellular HA in different cell types is also supported by literature data: intracellular localization of HA has been found to be associated with the rough ER [33], with heterochromatin and nuclei [34], with caveolae [35] and with cytoplasm [36]. Recent findings have also reported the presence of intracellular HA anchored to the cytoskeleton, being involved in mechano-transduction mechanisms [37].

Experiments based on the use of HA-Fl allowed us to assume that the signals derived from exogenously supplied HA and intracellular HA pools did not overlap since the fluorescein signal was restricted to large endosomal vesicles. This result, in addition to the previous experiment related to HA absence along the endocytic pathway, seemed to definitively contrast the hypothesis that the intracellular HA derived exclusively from the internalization of an endocytosed extracellular pool that would require translocation of the endosomal system from the lumen to the cytosol, leading to speculation of cytosolic synthesis at the inner face of the plasma membrane.

The enzymes responsible for synthesis, also called HA synthases (HAS1, HAS2, HAS3), are glycosyltransferases exerting their catalytic activity on the inner side of the plasma membrane. Mammals express three different isoforms which differ in the range and quantity of HA oligomers as well as in their enzymatic activity: HAS1 and HAS2 produce high molecular weight (HMW)-HA with an average size of 2 × 10^6^ Da, whereas HAS3 produces HA chains of smaller sizes that can reach at most 2 × 10^5^ Da [8]. HAS1 synthesizes lower amounts of HA than the other two HAS isoforms; in particular, HAS3 is the most catalytically active protein [38].

Although hyaluronan synthases are believed to be located exclusively in the plasma membrane [39], it is possible that, in the context of a tumor, they may be mislocalized on the intracellular membrane in a way that will end up secreting hyaluronan into the cytoplasm and/or nucleus of the cell. Dysregulation of HAS gene expression has been observed in cancer, with the pattern of expression tightly correlated to the cancer type [40]. Based on quantitative real-time PCR, we found that *HAS1* and *HAS2* are the isoforms predominantly expressed in MPM cells, with *HAS3* mRNA only poorly represented. These data are in accordance with the findings of Kanomata and colleagues, who reported that 90% of mesothelioma cases tested in IHC extensively immunoreacted to anti-HAS1 and HAS2 antibodies, while HAS3 overexpression was evident only in 40% of the cases [41]. Interestingly, when evaluating HAS espression at the protein level, we were able to detect consistent expression of all three isoforms both at the tissue and the cell level. This may be due to the poor correlation between the mRNA and protein expression levels usually observed in complex biological samples [42].

Another key component of the tumor microenvironment abundantly expressed in several type of cancers, where it can exert either tumor-promoting functions or anti-tumorigenic roles [19,43], is represented by the first subcomponent of the complement system, the C1q molecule. Since we previously demonstrated that C1q bound to HA is able to modify the signaling properties of the ECM enhancing adhesion, spreading and proliferation of MPM primary cells [20], we attempted to determine whether C1q and HA, either alone or in combination, may differentially impact HA homeostasis by altering the expression levels of the enzymes involved in its synthesis. Based on quantitative RT-PCR, we observed that only C1q bound to HA, but not C1q alone, was able to significantly enhance *HAS3* expression. This effect was selective for *HAS3*, since no significant modulation was observed for *HAS1* and *HAS2*, and these results were further confirmed at the protein level, both by cytofluorimetric and Western blot analyses. We may hypothesize that the modulation could be displayed only when C1q and HA were bound in a matrix because the binding was able to provoke a conformational change of C1q, altering the signaling properties of the tumor microenvironment and rendering it even more permissive to cancer survival, progression and migration through the promotion of HA synthesis.

Interestingly, bioinformatical analysis by PROGgene V2 database allowed us to unveil that high *HAS3* expression levels negatively correlate with survival expectancy in MPM patients. These observations appeared to be particularly reasonable since HAS3 has been addressed as the HAS enzyme more active and responsible for the production of smaller HA fragments, which are commonly associated with metastatic behaviors by promoting proliferation and invasion [44]. Since its low mRNA expression levels are reasonably offset by elevated protein expression and enzymatic activity as compared to other HAS [45] and since it exerts a significant role in cancer progression enriching the tumor microenvironment with pro-inflammatory and pro-tumorigenic HA fragments, HAS3 could be considered a future potential target in therapeutic MPM intervention.

## 4. Materials and Methods

### 4.1. Reagents and Antibodies

HA was a kind gift from Prof. Ivan Donati, Department of Life Sciences, University of Trieste. The following antibodies were used: mouse monoclonal anti-vimentin, mouse monoclonal anti-LAMP1, mouse monoclonal anti-HAS2 (#sc-365263) and mouse monoclonal anti-actin purchased from Santa Cruz Biotechnology (Santa Cruz, CA, USA); mouse monoclonal anti-EEA1, mouse monoclonal anti-Rab11A, mouse monoclonal anti-calnexin, mouse monoclonal anti-TGN38, streptavidin Cy3-conjugated and anti-mouse IgG fluorescein isothiocyanate (FITC)-conjugated from Abcam (Abcam Inc, Toronto, ON, Canada); rabbit polyclonal anti-C1q from Dako (Dako, Glostrup, Denmark); rabbit polyclonal anti-HAS1 (#PA5-50674) from Invitrogen^TM^ (Thermo Fisher Scientific, Waltham, MA, USA); rabbit polyclonal anti-HAS3 (#15609-1-AP) from Proteintech (Proteintech, Rosemount, IL, USA); anti-mouse LI-COR IRDye 680RD and anti-rabbit LI-COR IRDye 800CW from LI-COR Biosciences (LI-COR Biosciences, Lincoln, NE, USA). All chemicals were purchased from Sigma (Sigma-Aldrich, Saint Louis, MO, USA).

### 4.2. Patients and Specimens

Patients enrolled in the current study attended the Department of Pneumology, University Hospital of Cattinara, Trieste, Italy, with a symptomatic picture suggestive of MPM. Patients with reported asbestos exposure underwent video-assisted thoracoscopy, also known as pleuroscopy, for diagnosis of pleural effusion as previously described [20]. The eight selected patients were all male, Caucasian, presenting epithelioid histotype; none of them received chemotherapy or radiotherapy prior to sampling. The mean age at diagnosis was 76.4 ± 5.1 years.

All patients agreed to sign an informed consent form, following ethics approval by the Comitato Etico Unico Regionale (CEUR, Friuli Venezia Giulia, Italy; number 34/2016).

### 4.3. Cell Isolation and Culture

MPM cells were isolated from pleural biopsies of ten enrolled patients following the previously reported procedure [20]. MPM primary cells were cultured in a Human Endothelial Serum Free Medium (HESFM, Gibco) supplemented with 20 ng/mL epidermal growth factor (Sigma), 10 ng/mL basic fibroblast growth factor (Immunological Sciences), 1% penicillin–streptomycin (Sigma-Aldrich) and 10% heat-inactivated fetal bovine serum (FBS, Gibco). The cells were maintained at 37 °C in humidified atmosphere with 5% *v*/*v* CO_2_ and the medium was changed every 2–3 days. To assess the purity of isolated primary cells, they were characterized both by cytofluorimetric analysis and immunofluorescence assays for the following MPM representative markers: mesothelin, calretinin, cytokeratin 8/18, WT1 and CD44 in addition to CD45 and vWF to exclude leukocyte and endothelial cell contamination, respectively, as previously described [20].

Met5A cells, a non-malignant human pleural mesothelial line, were purchased from the American Type Culture Collection (ATCC) and cultured in complete HESFM. NCI-H28 cells, a human mesothelioma cell line, were purchased from the ATCC and cultured in the RPMI-1640 medium supplemented with 10% FBS.

### 4.4. Red Blood Cell Exclusion Assay

Met5A and MPM primary cells were seeded onto a sterile 96-well plate for cell culturing. The cells were grown in a culture medium until 70% confluence was reached. The medium was then removed and replaced by 0.16% fixed RBCs (Institut de Biotechnologies Jacques Bay) diluted into dPBS + 0.5% BSA, 0.7 mM CaCl_2_, 0.7 mM MgCl_2_. RBCs were allowed to settle down for 20 min at 37 °C in a 5% *v*/*v* CO_2_ incubator.

As a control, MPM primary cells were treated with recombinant hyaluronidases (Sigma) before the addition of the erythrocytes. Hyaluronidase treatment was performed as follows: the enzyme resuspended in 0.02 M phosphate buffer, pH 7, 77 mM sodium chloride, 0.01% BSA, was diluted to 500 units/mL in HESFM + 10% FBS, pH 4.41, and added to the cells for 30 min at 37 °C in a 5% *v*/*v* CO_2_ incubator.

Images were acquired by an optical microscope, original magnification 200×, with a Canon Power Shot A640 Camera.

### 4.5. Alcian Blue Staining

Tissue samples of MPM were fixed in 10% buffered formalin, paraffin-embedded and stored at 4 °C. Paraffin was steadily removed and sections were rehydrated by washing them in xylene, 100% EtOH, 95% EtOH, 70% EtOH and distilled water. Tissue sections were incubated with a solution of 1% Alcian blue dissolved in 3% acetic acid, pH 2.5, for 30 min at RT. After washing them in tap water for 10 min, the sections were dehydrated, and the nuclei were stained with Nuclear Fast Red.

MPM primary cells were seeded onto glass coverslips and allowed to grow to up to 70% of confluence. The cells were fixed with ice-cold methanol for 10 min at −20 °C and stained with 1% Alcian blue following the procedure described above. MPM primary cells were also treated with recombinant hyaluronidases (Sigma) as previously described. Cell nuclei were stained with hematoxylin. The slides were examined under a Leica DM 3000 optical microscope and images were acquired using a Leica DFC320 digital camera (Leica Microsystems, Wetzlar, Germany).

### 4.6. Immunofluorescence Microscopy for the Detection of HA

MPM primary cells in the amount of 5 × 10^4^ were seeded on round coverslips and allowed to grow to up to 70% of confluence. The cells were fixed with 2% paraformaldehyde (PFA) for 20 min at RT in the dark. Permeabilization, quenching and blocking were performed by incubating the cells in 1% BSA, 0.1% Triton X-100 and 50 mM glycine in dPBS for 30 min at RT. Incubation with primary antibodies and 5 μg/mL HABP (Merck Millipore) diluted with 3% BSA in dPBS was carried out ON at 4 °C. The following day, incubation with streptavidin and secondary antibodies (1:300) was performed for 30 min at RT. Nuclei were stained with DAPI (Sigma-Aldrich, 1:1000) for 5 min. The glasses were mounted with the Fluorescence Mounting Medium (Dako). Images were acquired using a confocal microscope Nikon Eclipse Ti2, SISSA facility.

In colabeling experiments, the following antibodies were used together with HABP: α-vimentin (1:40), α-calnexin (1:1000), α-Rab11a (1:1000), α-EEA1 (1:200), α-LAMP1 (1:50), α-TGN (1:100).

### 4.7. Detection of Endocytosed HA

MPM primary cells in the amount of 5 × 10^4^ were seeded on round coverslips and allowed to grow to up to 70% of confluence. HA-Fl (Sigma) in the amount of 10 μg/mL was added directly into the culture medium to be taken up during ON incubation at 37 °C in a 5% *v*/*v* CO_2_ incubator. The following day, the cells were fixed with 1% PFA for 20 min at RT in the dark. Permeabilization, quenching and blocking were performed by incubating the cells in 1% BSA, 0.1% Triton X-100 and 50 mM glycine in dPBS for 30 min at RT. MPM cells were then labeled with 5 μg/mL of HABP diluted in 3% BSA in dPBS for 1 h at RT. Incubation with streptavidin (1:300) was performed for 30 min at RT in the dark. The nuclei were stained with DAPI (Sigma-Aldrich, 1:1000) for 5 min. Coverslips were placed on a glass slide with the Fluorescence Mounting Medium (Dako).

Images were acquired using a confocal microscope Nikon Eclipse Ti2, SISSA facility.

### 4.8. Immunohistochemical Analysis

MPM tissue samples were fixed in 10% *v*/*v* buffered formalin and then paraffin-embedded. Tissue sections (4 µm-thick) were deparaffinized and rehydrated. The antigen unmasking technique was performed using EDTA-based Novocastra Epitope Retrieval Solutions (Leica Biosystems), pH 6, in a thermostatic bath at 98 °C for 30 min. The sections were then brought to room temperature and washed in PBS. After neutralization of the endogenous peroxidase with 3% *v*/*v* H_2_O_2_ and Fc blocking by a specific protein block (Novocastra, Leica Biosystems), the samples were incubated ON at 4 °C with the HAS1, HAS2, HAS3 and C1q primary antibodies. Staining was revealed using a polymer detection kit (Novocastra, Leica Biosystems) and the AEC (3-amino-9-ethylcarbazole, Dako, Denmark) or DAB (3,3′-diaminobenzidine) substrate chromogen. The slides were counterstained with Harris Hematoxylin (Novocastra, Leica Biosystems).

### 4.9. Flow Cytometry

MPM primary cells in the amount of 5 × 10^5^ were fixed in 3% PFA in the dark for 20 min and incubated with the anti-HAS1, HAS2 and HAS3 primary antibodies diluted in the permeabilization reagent of the FIX & PERM kit (Invitrogen, Life Technologies, Carlsbad, CA, United States) for 1 h on ice. Incubation with FITC-conjugated secondary antibodies was performed for 30 min on ice in the dark. The cells were resuspended and fixed in 1% paraformaldehyde.

Fluorescence was acquired with the FACScalibur (BD Bioscience) and the data were processed using the CellQuest software.

### 4.10. Coating Conditions

Cell culture plates were incubated ON at 4 °C with HMW-HA (MW, 1.5 MDa) at a concentration of 50 µg/mL in a bicarbonate buffer, pH 9.6. The following day, the plate was washed with dPBS and then incubated ON at 4 °C or for 2 h at 37 °C with C1q (Sigma) at a concentration of 25 µg/mL in dPBS + BSA 0.5% (Sigma), 0.7 mM CaCl_2_ and 0.7 mM MgCl_2_. Then, the wells were washed again with dPBS before seeding the cells.

### 4.11. Gene Expression Analysis

MPM primary cells in the amount of 10^6^ were seeded onto HA- or HA+C1q-coated 6-well plates and collected after ON incubation at 37 °C in a 5% *v*/*v* CO_2_ incubator. An untreated sample was also collected. The cell pellet was washed with ice-cold dPBS and centrifuged at 250× *g* for 7 min. After centrifugation, the cell pellet was resuspended into the RNA Lysis Buffer (EuroGOLD) and mRNA was isolated from cell lysates using a EuroGOLD Total RNA kit (EuroClone). Isolated mRNA was then retrotranscripted into cDNA using a SensiFAST cDNA Synthesis Kit (Bioline).

Quantitative real-time PCR was performed to investigate mRNA expression levels of HAS enzymes using a SYBR Select Master Mix (Applied Biosystems, Life Technology). The reaction was performed using Rotor-Gene 6000 (Corbett, Explera) following a program of 45 cycles of denaturation (60 s at 95 °C), annealing (30 s at 60 °C) and amplification (60 s at 72 °C).

Expression levels of the genes of interest were evaluated using comparative quantification based on the reaction efficiency and normalized for the concentration of a housekeeping gene, TATA box-binding protein (TBP), which is constitutively expressed in tumor cells. Primers used for quantitative real-time PCR analysis are reported in Table 1.

### 4.12. Western Blot Analysis

MPM primary cells in the amount of 10^6^ were seeded onto HA- or HA+C1q-coated 6-well plates and collected after ON incubation at 37 °C in a 5% *v*/*v* CO_2_ incubator. An untreated sample was also collected. Cells lysates were fractioned by 10% SDS-PAGE under reducing conditions and transferred to a nitrocellulose membrane using a semi-dry transfer apparatus Trans-Blot Turbo System according to the manufacturer’s protocol (BIO-RAD). After 1 h of incubation with 5% skim milk in TBST (10 mM Tris, pH 8.0, 150 mM NaCl, 0.5% Tween 20), the membrane was probed with anti-HAS3 and anti-actin antibodies ON at 4 °C. Membrane was washed three times for 5 min and incubated with LI-COR IRDye secondary antibodies for 1 h at RT. After three washing steps, fluorescence intensity was identified using an Odyssey^®^ CLx near-infrared scanner (LI-COR Biosciences, Lincoln, NE, USA). Image acquisition, processing and data analysis were performed with Image Studio ver 5.2 (LI-COR Biosciences).

### 4.13. Bioinformatical Analysis

Survival analyses were performed with PROGgeneV2 (www.genomics.jefferson.edu/proggene/index.php) using data from The Cancer Genome Atlas Mesothelioma (TCGA-MESO) dataset [46,47]. All the results were displayed with *p*-values from a log-rank test; *p* < 0.05 was considered significant.

### 4.14. Statistical Analysis

The data were analyzed using unpaired one-tailed Student’s *t*-test. Results were represented as the means ± SEM; *p* < 0.05 was considered statistically significant. All statistical analyses were performed using Prism 5.0 software (GraphPad Software Inc., La Jolla, CA, USA).

## 5. Conclusions

In conclusion, isolated MPM primary cells were able to synthesize HA by themselves by creating a pericellular coat and extracellular protrusions, as well as by maintaining an intracellular pool mainly localized in the cytoplasm and in the perinuclear region. Moreover, HA synthesis was modulated by the influence of the C1q–HA matrix, which, acting as a pro-tumorigenic signaling complex, was able to significantly increase HAS3 expression and consequently enhance production of HA fragments, allowing the identification of a future potential target in MPM.

## Figures and Tables

**Table 1:** Primers used for quantitative real-time PCR.

Gene	Melting Temperature	Forward Sequence Reverse Sequence	Accession Number
*HAS1*	60	GGAATAACCTCTTGCAGCAGTTC TCATCCCCAAAAG	NM_001523.3
*HAS2*	58	TCGCAACACGTAACGCAAT ACTTCTCTTTTTCCACCCCATTT	NM_005328.2
*HAS3*	60	CGCAGCAACTTCCATGAGG AGTCGCACACCTGGATGTAGT	NM_005329.2
*TBP*	60	GAGCCAAGAGTGAAGAACAGTC GCTCCCCACCATATTCTGAATCT	NM_003194.4

## Data Availability

The data presented in this study are available within the article and Supplementary Materials.

## Supplementary Materials

The following are available online at https://www.mdpi.com/2072-6694/13/3/416/s1, Figure S1: HASes modulation in MPM cells after treatment with soluble C1q and/or HA. MPM cells were treated with soluble C1q and/or HA and analyzed for the expression of HAS1 (**A**), HAS2 (**B**) and HAS3 (**C**) through quantitative Real-Time PCR, showing no statistically significant differences. TBP was used as a housekeeping gene to normalize gene expression results.
