# Supplementary material for: C1q–HA Matrix Regulates the Local Synthesis of Hyaluronan in Malignant Pleural Mesothelioma by Modulating HAS3 Expression
Source: Cancers (Basel). 2021 Jan 22;13(3):416. doi: 10.3390/cancers13030416 (PMC7865933; doi:10.3390/cancers13030416)
Supplement: Supplementary file 1 [file cancers-13-00416-s001.pdf]

cancers

MDPI

Article

C1q–HA Matrix Regulates the Local Synthesis of Hyaluronan in Malignant Pleural Mesothelioma by Modulating HAS3 Expression

Romana Vidergar [1,†], Andrea Balduit [1,†,*], Paola Zacchi [1], Chiara Agostinis [2], Alessandro Mangogna [2], Beatrice Belmonte [3], Micaela Grandolfo [4], Francesco Salton [5], Marco Biolo [5], Fabrizio Zanconati [5], Marco Confalonieri [5] and Roberta Bulla [1]

Citation: Vidergar, R.; Balduit, A.; Zacchi, P.; Agostinis, C.; Mangogna, A.; Belmonte, B.; Grandolfo, M.; Salton, F.; Biolo, M.; Zanconati, F.; et al. C1q-HA Matrix Regulates the Local Synthesis of Hyaluronan in Malignant Pleural Mesothelioma by Modulating HAS3 Expression. *Cancers* **2021**, *13*, 416. https://doi.org/10.3390/10.3390/cancers13030416

Academic Editor: Giulia M Stella; Chandra Bortolotto

Received: 30 November 2020
Accepted: 20 January 2021
Published: 22 January 2021

Publisher's Note: MDPI stays neutral with regard to jurisdictional claims in published maps and institutional affiliations.

Copyright: © 2021 by the authors. Submitted for possible open access publication under the terms and conditions of the Creative Commons Attribution (CC BY) license (http://creativecommons.org/licenses/by/4.0/).

1 Department of Life Sciences, University of Trieste, 34127 Trieste, Italy; romana_vidergar@immunol.a-star.edu.sg (R.V.); pzacchi@units.it (P.Z.); rbulla@units.it (R.B.)
2 Institute for Maternal and Child Health, IRCCS Burlo Garofolo, 34134 Trieste, Italy; cagostinis@units.it (C.A.); alessandro.mangogna@burlo.trieste.it (A.M.)
3 Tumor Immunology Unit, Department of Health Sciences, University of Palermo, 90133 Palermo, Italy; beatrice.belmonte@unipa.it
4 International School for Advanced Studies (SISSA), 34136 Trieste, Italy; micaela.grandolfo@sissa.it
5 Department of Medical, Surgical and Health Science, University of Trieste, 34129 Trieste, Italy; francesco.salton@studenti.units.it (F.S.); marco.biolo@asugi.sanita.fvg.it (M.B.); fabrizio.zanconati@aots.sanita.fvg.it (F.Z.); marco.confalonieri@asugi.sanita.fvg.it (M.C.)
* Correspondence: abalduit@units.it
† These authors contributed equally.

Supplementary

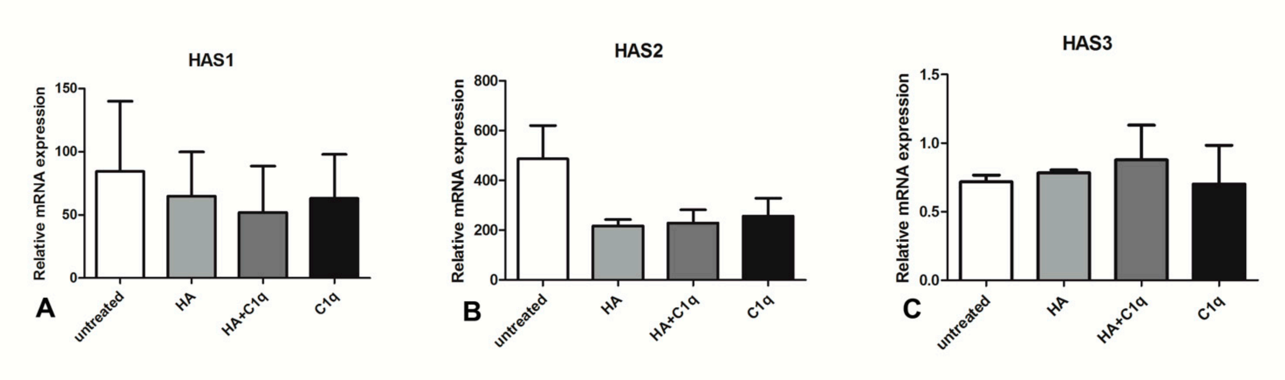

Figure S1. HASes modulation in MPM cells after treatment with soluble C1q and/or HA. MPM cells were treated with soluble C1q and/or HA and analyzed for the expression of HAS1 (**A**), HAS2 (B) and HAS3 (C) through quantitative Real-Time PCR, showing no statistically significant differences. TBP was used as a housekeeping gene to normalize gene expression results.
